# Understanding and improving access to prompt and effective malaria treatment and care in rural Tanzania: the ACCESS Programme

**DOI:** 10.1186/1475-2875-6-83

**Published:** 2007-06-29

**Authors:** Manuel W Hetzel, Nelly Iteba, Ahmed Makemba, Christopher Mshana, Christian Lengeler, Brigit Obrist, Alexander Schulze, Rose Nathan, Angel Dillip, Sandra Alba, Iddy Mayumana, Rashid A Khatib, Joseph D Njau, Hassan Mshinda

**Affiliations:** 1 Department of Public Health and Epidemiology, Swiss Tropical Institute, P.O. Box, CH-4002 Basel, Switzerland; 2 Ifakara Health Research and Development Centre, P.O. Box 53, Ifakara, Tanzania; 3 Novartis Foundation for Sustainable Development, WRO-1002.11.56, CH-4002 Basel, Switzerland

## Abstract

**Background:**

Prompt access to effective treatment is central in the fight against malaria. However, a variety of interlinked factors at household and health system level influence access to timely and appropriate treatment and care. Furthermore, access may be influenced by global and national health policies. As a consequence, many malaria episodes in highly endemic countries are not treated appropriately.

**Project:**

The ACCESS Programme aims at understanding and improving access to prompt and effective malaria treatment and care in a rural Tanzanian setting. The programme's strategy is based on a set of integrated interventions, including social marketing for improved care seeking at community level as well as strengthening of quality of care at health facilities. This is complemented by a project that aims to improve the performance of drug stores. The interventions are accompanied by a comprehensive set of monitoring and evaluation activities measuring the programme's performance and (health) impact. Baseline data demonstrated heterogeneity in the availability of malaria treatment, unavailability of medicines and treatment providers in certain areas as well as quality problems with regard to drugs and services.

**Conclusion:**

The ACCESS Programme is a combination of multiple complementary interventions with a strong evaluation component. With this approach, ACCESS aims to contribute to the development of a more comprehensive access framework and to inform and support public health professionals and policy-makers in the delivery of improved health services.

## Background

The impact of malaria on health and local economies in sub-Saharan Africa is staggering. Between one and three million people die each year, mostly young children under five years of age. Deaths and illness contribute to a vicious circle of ill-health and poverty [1,2]. In recent years, the fight against malaria has gained an increased level of attention from governments of affected African states as well as from international donor agencies. African heads of state agreed in the Abuja Declaration on a concerted effort to reduce the burden of malaria on the continent and endorsed the ambitious goal of the Roll Back Malaria Partnership of halving the number of malaria deaths by the year 2010 [3]. Among the malaria control strategies promoted internationally and adopted by most endemic African countries, prompt access to effective treatment especially for young children and pregnant women features prominently [2].

The need for prompt and effective treatment to prevent progression to severe disease and death essentially raises two important issues: first, the choice of a safe and efficacious drug and second, questions of how to optimize equitable access to rationally prescribed treatment.

In order to address the first point, artemisinin-based combination therapies (ACT) have been advocated as treatment of choice in Africa [4] in an effort to improve on drug efficacy following the increasing failure rate of a number of other drugs. Tanzania adopted this policy in 2004 and implemented it at the end of 2006 [5]. However, the choice of an efficacious drug does not necessarily directly result in improved effectiveness, and issues related to safety, use in pregnancy, and cost are also still being discussed. Yet, it would go beyond the scope of this paper to thoroughly debate all issues related to a specific drug.

With regard to the second point, it is widely acknowledged that access to quality treatment is insufficient in many settings. The poorest people often have least access to effective treatment [6] and the underlying causes of this situation are increasingly debated. On a macro-level, the discussion on access to treatment often focuses around the development of new drugs [7] and global affordability issues, including pricing and patenting of drugs. International initiatives, such as Medicines for Malaria Venture (MMV) [8], are increasingly financing and speeding up the development and introduction of new efficacious antimalarials. At a local community level however, the situation is a lot more complex and availability and affordability of drugs are only few among a number of factors influencing prompt and effective treatment [9,10]. In many developing countries, weak health systems as well as lack of equipment and qualified staff lead to incorrect diagnosis and treatment [11,12]. Physical access may be impeded by long distances to the nearest point of care, inadequate logistics or inability to pay for secondary costs such as transport [13]. Further, malaria is a common and socially well accepted illness in endemic countries and its potential severity is often underestimated. Insufficient knowledge of the appropriate treatment or an understanding of the illness that differs from the bio-medical explanation can lead to the use of alternative treatment sources and non-adherence to recommended regimens [14,15].

Several initiatives have attempted to address access questions on a local level, either by strengthening home-based management [16], by improving the involvement of commercial drug providers [17] or through a general improvement of health system performance. Information and education of caretakers and care providers has been useful in improving malaria case management and compliance at home and in drug selling shops [17-19]. Several models for improving case-management in health facilities have been tested and combined approaches were most likely to have a (sustainable) impact [20]. In any case, considering the complexity of the issues involved it seems obvious that there is no such thing as a single "magic bullet" approach to solve the problem. What is needed is a comprehensive concept addressing several of the access dimensions, ranging from availability and affordability to accessibility, acceptability and quality of care. This paper presents a programme that was developed to understand and improve comprehensively access to appropriate malaria treatment in a highly malaria-endemic rural area of south-eastern Tanzania.

The aim of the ACCESS Programme is to investigate factors influencing access to malaria treatment in rural Tanzania in order to develop a set of interventions addressing the main obstacles to access. These interventions are then thoroughly evaluated. The focus is on children below five years of age and pregnant women, who are the most vulnerable groups in this holo-endemic setting in terms of the detrimental consequences of malaria [21,22]. This paper presents a general overview of the ACCESS Programme, while future reports will provide detailed study results of the major evaluation and monitoring components.

## Project description

### Study area

**Figure 1:**
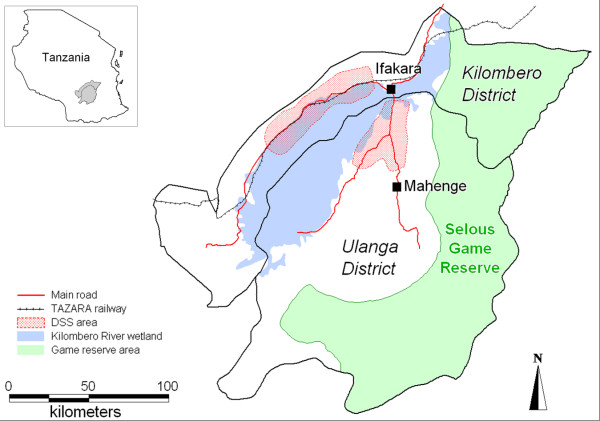
Map of Kilombero and Ulanga districts with DSS area.

The programme's intervention area comprises the two districts of Kilombero and Ulanga in the south-east of Tanzania (Figure 1). The Kilombero River separates the two districts and forms the vast Kilombero Valley floodplain. Large parts of this valley are flooded during the rainy season which usually lasts from November to May. The valley is delimited by the Udzungwa Mountains in the north and the Mahenge Mountains in the south. Parts of Ulanga's southern and south-eastern areas, as well as Kilombero's extreme east are part of the Selous Game Reserve. The Kilombero district is connected to the Tanzania-Zambia highway through a mostly unpaved but well maintained road. For vehicles the only connection to the Ulanga district is made by a motorized ferry over the Kilombero River.

In 2002, there were 517,000 people living in the 109 villages of the two districts. Ifakara, the administrative capital of Kilombero, is the major settlement in the valley with a population of approximately 46,000. Ulanga's capital Mahenge is smaller with 7,300 inhabitants [23]. In the early 1970s, the national social engineering project to build communal villages ("vijiji vya ujamaa") brought the valley's scattered inhabitants to more organised village centres along the edges of the valley [24]. Most people there rely on subsistence farming for their livelihoods. Labour intensive rice farming on distant fields in the floodplain forces many families to move to their farming sites (*shamba *in Kiswahili) during the cultivation period. In the fields people stay in improvised "*shamba *huts" for up to six or more months. Rice, maize and cassava are the main cash crops. The main agricultural exports from both districts are rice, timber, charcoal and some fish. Since the 1980s, an increasing number of nomadic Maasai and Sukuma pastoralists had moved to the valley with large cattle herds until a government directive ordered them in April 2006 to move to other parts of the country or to reduce their herds in order to preserve the Kilombero wetland ecosystem [25,26].

The climatic and ecological conditions in the floodplain are favourable for mosquito breeding. Malaria transmission in the valley is high and perennial. Recent work has confirmed entomological inoculation rates (EIR) of 350 infective bites per person per year, despite high mosquito net usage rates of 75% (G. Killeen, personal communication). At the level of health services, malaria is the most frequent diagnosis in outpatients in rural health facilities.

A number of malaria control interventions have been tested and/or implemented in the area by the Ifakara Health Research and Development Centre [27] in collaboration with the Swiss Tropical Institute [28]. The most extensive operation was the large-scale introduction of insecticide-treated nets (ITN) in the frame of the KINET project [29]. Today, promotion of ITN use through social marketing is ongoing in the frame of the national ITN programme.

Monitoring and evaluation (M&E) of the ACCESS Programme is carried out in the area of the local Demographic Surveillance System (DSS) [30]. The DSS serves as a comprehensive epidemiological framework for studies on the project's impact. In the absence of a vital registration, DSS field workers routinely record births, deaths, migrations and socio-economic indicators for every household in a defined geographical area of 2400 km^2^(Figure 1). Each household is visited every four months. The area comprises 12 villages in Ulanga and 13 in Kilombero. In mid 2004, the total DSS population was 74,200 (Ulanga: 31,800; Kilombero: 42,400) in 17,050 households. The DSS does not include Ifakara town where ACCESS M&E activities are also implemented.

### Main interventions

ACCESS interventions apply two main approaches:

1. Creating demand for appropriate malaria diagnosis and treatment in the community through a social marketing approach.

2. Strengthening the supply of quality malaria case-management at health facilities and drug shops through training, quality management, improved supportive supervision and new diagnostics.

**Figure 2:**
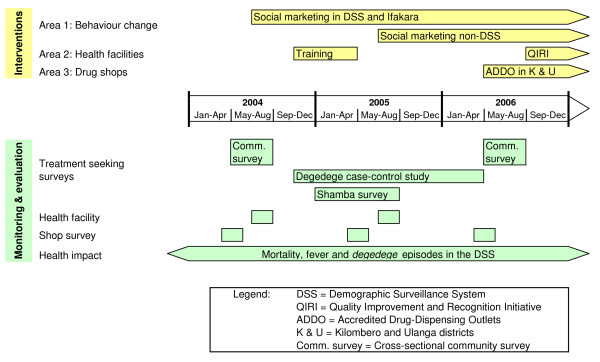
Timeline with main interventions and M&E activities.

The main areas of intervention are described below and summarized in Figure 2 (status at the end of 2006). Activities may change in the future as experience is gathered and analyzed by the programme's M&E activities.

### Intervention area 1: Behaviour change campaigns for prompt and appropriate health care seeking

#### Sensitization of community leaders

As a first preparatory step in implementing community activities, local community leaders (political and religious leaders, leaders of social groups, non-governmental organizations and other key opinion leaders) were informed about ACCESS objectives and activities to gain their support and collaboration. The meetings also provided space for participants to share their views and concerns on programme-related issues.

#### Social marketing

A social marketing approach was chosen to increase knowledge and awareness of malaria and to promote prompt and appropriate treatment seeking from reliable sources. The design of the behaviour change communication campaign was based on experiences from projects such as KINET (Kilombero Net Project) [29,31], TEHIP (Tanzania Essential Health Interventions Project) [32] and IMPACT-Tz (Interdisciplinary Monitoring Project for Antimalarial Combination Therapy in Tanzania), as well as the national social marketing for ITNs and the results from exploratory focus-group discussions. In the first year, implementation was done in the DSS area and Ifakara only, followed by a step-wise scaling-up to the other villages of Kilombero and Ulanga.

The main *target audience *of the campaign are mothers and caretakers of children under five years of age and pregnant women. However, other household members and the general population are secondary audiences in order to achieve homogeneity of understanding in the population.

*Messages *stress the importance of prompt recognition of malaria symptoms and immediate correct treatment with the recommended first-line drug (sulphadoxine-pyrimethamine [SP] until end 2006). Health facilities and licensed drug stores (pharmacies, part II drug stores [*duka la dawa baridi*] and Accredited Drug Dispensing Outlets [ADDO; *duka la dawa muhimu*]) are promoted as sources of proper treatment and advice. Prevention methods, such as the use of ITNs and Intermittent Preventive Treatment in pregnancy (IPTp) are also advocated. Finally, one set of messages highlights high fever with convulsions (locally known as *"degedege"*) as a sign of severe malaria that can and should be treated at health facilities (rather than by traditional healers) [14,31]. ACCESS messages are in line with malaria-related messages on key family practices of the community-based Integrated Management of Childhood Illness (IMCI) [33].

**Figure 3:**
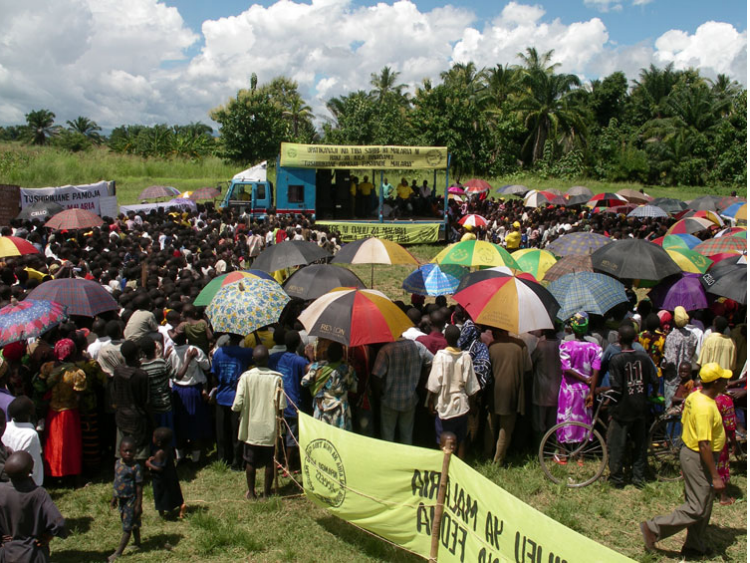
ACCESS road show with social marketing truck.

*Communication channels *and materials to disseminate behaviour change messages were developed to reach a poor rural population in an efficient and cost-effective way. Road shows are the main vehicle for the campaign. The platform of a truck is used as a mobile stage for a health promotion team (Figure 3). The shows are divided in four parts:

1. Dancing competition to attract a large audience

2. Comedies and role plays portraying appropriate treatment seeking and consequences of delaying treatment

3. Public lecture on malaria transmission, signs and symptoms, dangers of malaria for young children and pregnant women, and prevention and correct treatment

4. Cinema show featuring stories on prompt and effective malaria treatment

Question-and-answer sessions at the end of each part allow interaction with the audience and distribution of promotion-materials (e.g. stickers, leaflets, T-shirts).

**Figure 4:**
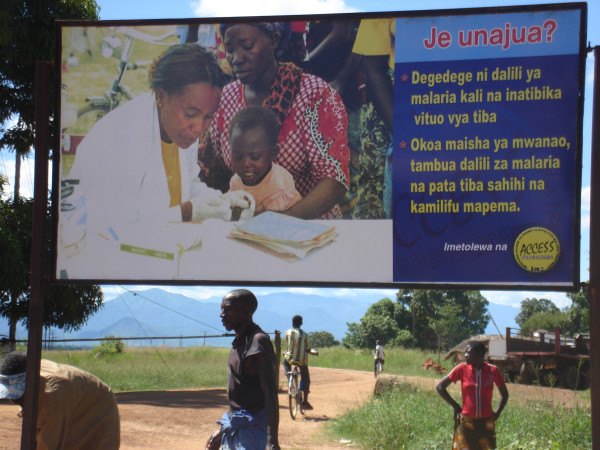
ACCESS billboard promoting prompt and correct treatment.

**Figure 5:**
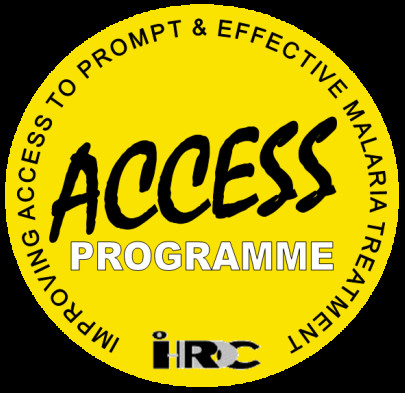
ACCESS Programme logo.

Permanent billboards were erected in major villages along the main road and posters affixed in public places (Figure 4). All materials carry campaign-related messages and the ACCESS logo (Figure 5). Materials were locally designed and pre-tested in the community.

Remote villages which are inaccessible with the truck are reached by a small 4WD vehicle branded with behaviour change slogans. It transports a mobile video unit and rooftop speakers to air behaviour change radio spots.

#### Special campaigns in Mother and Child Health (MCH) clinics

Special campaigns were implemented in MCH clinics. They were targeted especially at pregnant women and mothers of young children who may not attend road shows if they overlap with the women's duties in the household. During special sessions, ACCESS health promoters and MCH clinic staff informed mothers on malaria, its prevention and its proper treatment. The benefits of malaria prevention using ITNs and IPTp were particularly emphasized.

#### Improved access for households spending the cultivation period away from home: The shamba component

The main farming season, when many families stay in their field-huts, overlaps with the high malaria transmission season. Furthermore it represents a period of high vulnerability as it coincides with peak food insecurity, labour stress and difficult access to health services, family support and child care time. A study was undertaken within the programme to investigate the specific risks posed by staying in the fields. Results from this study may lead to the design of a "shamba intervention" if specific measures are deemed necessary.

### Intervention area 2: Improved quality of care in health facilities

Trustworthy health care services of good quality are a core element for the delivery of effective diagnosis and treatment for malaria. As a result of the social marketing, the demand for quality services is expected to increase. In order to meet this demand, the health facility staff must be in the position and willing to deliver a good quality of care. The programme aims to improve quality of care with a focus on the following areas:

• Correct diagnosis through the proper use of the IMCI algorithm or with improved laboratory diagnosis

• Rational prescription of antimalarials, antipyretics and other drugs

• Appropriate advice on prescribed treatment and malaria prevention

Key activities of this component include initial refresher training for health facility staff on malaria treatment, followed by the strengthening of routine supportive supervision and the implementation of a quality management scheme in all health facilities. Training was based on IMCI algorithms for diagnosis and treatment which have proven (cost-)effective in improving quality and efficiency of child health care in rural Tanzania [34]. A protocol for the refresher training was developed in close collaboration with the Council Health Management Teams (CHMT) of Kilombero and Ulanga. The training was targeted at clinical staff, lab technicians, and medical aids of public and private health facilities. It was carried out by the CHMT, appointed trainers and ACCESS staff with financial resources from the district and ACCESS.

The follow-up with routine supportive supervision will not focus on malaria only. It is planned to implement a comprehensive package of activities aiming at improving performance management for improvement of quality of services delivery: The Quality Improvement and Recognition Initiative (QIRI) was originally developed by USAID and adapted and implemented in Tanzania by the Ministry of Health (MOH) to improve the quality of reproductive and child health services within the 2001–2005 USAID-Tanzania programme support [35]. Between 2003 and 2006, the United Nations Population Fund (UNFPA) Tanzania through the European Union/UNFPA Sexual and Reproductive Health programme supported the MOH for further QIRI expansion in Tanzania [36].

QIRI offers an integrated approach for the evaluation of quality of care combined with a strategy to establish the root causes of performance gaps and to develop implementable strategies to address them. A central element of this component is capacity building for joint supportive supervision and quality management, conducted by the regional and district health management teams together with community representatives. It is the aim of the programme to integrate quality management into the health supervision activities in the decentralized health system. Acknowledging the importance of patient-provider relationships and trust in health care [37,38], QIRI is designed to pay particular attention to the patients' perception of the health services.

In most malaria endemic areas, diagnosis of malaria relies mainly on clinical signs and symptoms, especially in low level health facilities. In Tanzania, only hospitals and health centres are expected to have the possibility of performing microscopy for malaria diagnosis [39], while dispensaries rely on a syndromic IMCI approach [40]. In the programme area, the malaria-attributable fraction estimated using the method of Smith *et al. *[41] showed that only 40% of all fever episodes were likely to be due to malaria (S. P. Kachur and S. Abdulla, personal communication). Absence of lab diagnosis may result in misdiagnoses and irrational drug-prescription [11,42].

A promising alternative to microscopy are rapid diagnostic tests (RDT) based on the detection of *Plasmodium *antigens. However, there is little experience with RDTs in sub-Saharan Africa although they are widely used in Asia and Latin America [43]. ACCESS plans to introduce RDTs in three dispensaries, so far lacking diagnostic tools for malaria. To compare the feasibility and value of RDTs versus conventional diagnostics, high quality microscopy will be assured in two health centres. The efficacy, effectiveness and cost-effectiveness of this intervention will be evaluated.

### Intervention area 3: Improved malaria case management in drug selling shops

Self-treatment at home is often the first and quickest response to a malaria episode [16,44,45]. In many settings, the private drug retail sector plays an important role in providing drugs for home-based management of fever or malaria. On the other hand, drug shops often leave patients with sub-standard malaria drugs and poor prescribing practices [46], leading to ineffective treatment and increasing drug resistance. Experience in Kenya showed that training private drug retailers can considerably improve the services they offer [17]. However, Tanzanian drug regulations do not allow general shops to sell the first-line antimalarial drugs (SP; or ACT since end 2006), even though the national malaria control strategy mentions explicitly the availability of antimalarials on household-level [5,47,48].

As a result of this ambiguous policy, the initial plan to train general shop keepers had to be withdrawn and other avenues explored. As an alternative, the programme supports the introduction of Accredited Drug Dispensing Outlets (ADDO; *duka la dawa muhimu *in Kiswahili) in the two districts. The ADDO project is being implemented by the Tanzania Food and Drugs Authority (TFDA) and Management Sciences for Health (MSH). It aims to improve access to affordable, quality medicines and pharmaceutical services in drug retail outlets in rural and peri-urban areas where there are few or no registered pharmacies [49,50]. The main components of the ADDO project are activities to change the behaviour of shop owners and dispensing staff through the provision of education, incentives and regulatory coercion. It also entails efforts to positively affect client demand and expectation of quality products and services.

ADDOs are allowed to dispense a limited range of prescription-only medicines that are found on the national essential drugs list. Ideally at least one ACT should be available through this channel, most logically the one recommended as first-line treatment in the country (currently artemether/lumefantrine [ALu], brand name Coartem^®^). For the districts of Kilombero and Ulanga, ACCESS could successfully negotiate the introduction of highly subsidized ALu in ADDOs. The ACCESS social marketing campaign promotes ADDOs as source of quality malaria treatment.

### Monitoring and evaluation

The M&E activities of ACCESS are based on three key components: (1) A semi-quantitative analysis aiming at a better understanding of factors influencing access to malaria treatment in order to develop an improved access framework, (2) process monitoring in order to understand how interventions operate, and (3) a thorough evaluation of the programme's impact on treatment seeking, quality of case-management and most importantly on the health of the population. An overview of the different evaluation activities in relation to key work areas is given in Table 1.

**Table 1:** ACCESS Programme components and corresponding indicators for evaluation (refer to main text for details)

**Indicator**	**Sources of verification/study methods**	**Sample**	**Timing**
**Intervention area 1: Behaviour change campaign** (Expected results: Improved health care seeking behaviour for all fever/malaria episodes)			

Proportion of episodes treated according to national guidelines within 24 h Treatment-shift to qualified providers	Cross-sectional community surveys (fever and degedege) with EMIC tool	Random sample of households	Repeated (baseline, mid-term, end)
	DSS (morbidity) and health facility attendance	DSS area, all health facilities	Continuous
Equitable access to appropriate treatment	DSS SES data	DSS area, all health facilities	Continuous

**Intervention area 2: Quality of care in health facilities **(Expected results: Improved quality of care in health facilities, especially malaria case-management, incl. diagnosis, prescription, treatment, advice, compliance)			

Proportion of episodes receiving correct prescription and appropriate advice Patient's satisfaction with services	Quality of care surveys in health facilities	Sample of health facilities	Repeated (baseline, mid-term, end)
	QIRI	All health facilities	Continuous

**Intervention area 3: Malaria case-management in shops** (Expected results: Improved quality of malaria case-management in drug selling shop, such as retailing practices, prescriptions, advice)			

Proportion of episodes receiving correct prescription and appropriate advice	Mystery shoppers	All drug stores and random sample of general shops	Annually
Shop-keepers' knowledge of malaria symptoms, correct treatment and advice Availability of first- and second-line antimalarial drugs	Cross-sectional surveys in shops stocking drugs	All retailers stocking drugs	Annually

**Shamba component** (Expected results: Coverage of appropriate malaria treatment and care services extended to underserved areas, incl. shamba households)			

Proportion of households within 5 km range of qualified provider	DSS GPS data	All households, health facilities and shops stocking drugs	Repeated
Proportion of episodes in underserved areas/poor households/shamba houses receiving correct treatment	Cross-sectional community surveys (fever and degedege), DSS SES data	Random sample of households	Repeated (baseline, mid-term, end)

**Health impact** (Expected results: Reduction of malaria related morbidity and mortality, especially in children under five and pregnant women)			

Proportion of malaria-related deaths	DSS mortality data: overall and cause-specific.	All households	Continuous
Number of fever episodes.	DSS fever incidence	All households	Continuous

**Additional studies**			

Understanding and perception of malaria, its treatment and prevention	Focus-group discussions	10 groups of caretakers of children under five years in Ifakara and DSS	Once prior to interventions
Risk factors for fatal outcome of *degedege*	Case-control study	Cases:*degedege*-related child deaths in DSS Controls: recovered *degedege *cases	Once
Vulnerability and coping strategies of households during the farming season; movement patterns and health seeking	Cohort study with *shamba *households	Random sample of households with field (*shamba*) house	Once
Antimalarial drug quality	Cross-sectional survey	All drug stocking retailers and health facilities	Once

EMIC = Explanatory Model Interview Catalogue. DSS = Demographic Surveillance System. SES = Socio-economic Status. QIRI = Quality Improvement and Recognition Initiative. GPS = Geographic Positioning System.

Overall and health impact evaluation is based on a plausibility assessment of the programme's impact within a before-after design, i.e. a historical control group [51,52]. In an attempt to control for possible confounders, all other malaria control activities in both districts as well as other relevant parameters such as temperature and rainfall are closely monitored. Longitudinal data will also be compared to trends observed in Demographic and Health Surveys (DHS). A basic assumption is that the malaria transmission and other relevant epidemiological parameters remain largely unchanged during the period of observation with the exception of the factors that are monitored in the frame of the programme.

#### Health impact assessment through the DSS

The health impact assessment will be based on data collected through the DSS. The main outcome indicators are: overall and malaria-specific mortality, reported fever incidence rates in children and adults, as well as reported *degedege *(convulsion) rates in children. Furthermore, the DSS will be used as a sampling frame for representative community-based epidemiological studies.

Cause-specific mortality is calculated on the basis of "verbal autopsies". Since 2002, specially-trained DSS supervisors elicit information on causes of deaths by interviewing bereaved relatives about the circumstances of the death, the signs and symptoms observed during the illness leading to the death, and the action taken. This information is coded to give likely causes of death in broad categories.

The socio-economic status (SES) of households is assessed once a year on the basis of a list of household assets. This allows the results of DSS data and other community-based studies to be stratified by wealth quintiles, which is essential in order to consider equity dimensions in the analysis. The aim of the ACCESS Programme is to contribute to an equitable reduction of (malaria-related) mortality.

#### Exploratory focus-group discussions

Initial exploratory focus-group discussions (FGD) with parents and caretakers of young children informed the programme on knowledge, attitudes and practices related to malaria treatment. A total of 88 people participated in the ten FGDs, four of which were done with men, six with women. Main issues that came up during FGDs are listed in the results section.

#### Community-based surveys on treatment-seeking for fever

Repeated cross-sectional community surveys are the programme's main tool to assess changes in care-seeking behaviour for fever episodes. Explanatory Model Interview Catalogues (EMIC) are used to simultaneously collect cultural epidemiological qualitative and quantitative data on patterns of distress, perceived causes and help seeking [53]. A baseline survey was carried out in 2004 in the DSS villages and Ifakara town. Interviews were done with 80 caretakers of children and with 68 adults who experienced a fever episode in the preceding two weeks. Only people who had recovered from their illness the day of the interview were included while others were advised to consult a health professional. The same methodology will be applied in follow-up surveys every two years. It is expected that over the course of the programme, the number of appropriately treated fever episodes will increase with more people shifting to qualified care providers.

The EMIC was also used in a longitudinal study exploring treatment seeking during the cultivation period, when many people live in their *shamba *huts. About 100 household owning a temporary home in the fields were randomly sampled from DSS villages and followed up during one farming season. Each household was visited once a month by a team of field workers who recorded each family member's stay in the field, the occurrence of fever episodes and other indicators. In case of a fever episode in the preceding two weeks, an EMIC interview was conducted.

A household survey in a larger sample of 3'654 persons carried out in 2006 by a partner project (IMPACT-Tz) in the study area was used to assess uptake of social marketing messages by the population.

#### Case-control study on degedege

A case-control study on *degedege *(convulsions) was nested in the DSS data collection. The study compares treatment seeking patterns and self-observed signs and symptoms for fatal ("cases") and non-fatal ("controls") *degedege *episodes in children. *Degedege *has commonly been treated by traditional healers rather than with modern medicine [14]. However, this may have changed over time. EMIC questionnaires and extended verbal autopsies (VA) were used as data collection tools for non-fatal and fatal cases respectively. Non-fatal *degedege *cases were reported routinely by DSS field-workers. A random sample was then followed up every two weeks for an EMIC interview. This study is expected to provide information on observed "danger signs" and factors related to treatment seeking and leading to death or recovery. It will add an important aspect to the existing knowledge on management of fatal malaria including cases of convulsions as described by de Savigny *et al. *[54].

#### Quality of care at health facilities

Initial assessment of quality of care is based on yearly surveys in a sample of public and private/mission health facilities. Tools were adapted from the multi-country evaluation of IMCI [55]. Activities include patient-provider observations, as well as staff and patient exit interviews. Furthermore, laboratory equipment is checked for functionality and drug stocks are recorded. With the implementation of the QIRI tools for supportive supervision in 2007 (as outlined above), evaluation of quality of care will be done largely through QIRI which will assess quality of care twice annually in all health facilities. Results will then feed directly into activities aimed at improving quality of care. It is expected that the programme's interventions will lead to improved malaria case-management and more rational prescription of antimalarial drugs.

#### Health facility attendance and availability of antimalarials

Health facility attendance data and frequency of specific diagnoses are routinely recorded by health facility staff for the health management information system (HMIS). This information is collected bi-monthly from all private and public health facilities in Ifakara (one public, one private) and the DSS area (10 public, five private) by ACCESS staff. Together with the DSS fever incidence data which provides a community estimate, this information will allow to calculate the proportion of fever cases diagnosed as malaria and treated at health facilities. This proportion is expected to rise over the course of the project. In the frame of this activity, availability of antimalarial drugs is monitored in all health facilities in the DSS.

#### Quality of antimalarial drugs

In 2005, a study was designed to get an overview of the quality of antimalarials available in the programme's study area. For this purpose, all antimalarial selling points in the 25 DSS villages as well as in Ifakara were visited, including general shops, drug stores, pharmacies and health facilities. Samples of SP, amodiaquine and quinine products were purchased and the amount of active ingredient quantified according to the United States Pharmacopoeia (USP 24) using previously set up high-performance liquid chromatography (HPLC) methods [56,57]. In accordance with USP standards, products with less than 90% and more than 110% of the labelled amount of active ingredient were counted as failures.

#### Quality of services at shops selling drugs

Based on the methodology developed by Goodman *et al. *[58] for monitoring antimalarial drug availability, the DSS villages and Ifakara are searched annually for drug-selling shops and the shopkeepers are interviewed. A structured questionnaire is used to record current drug stock and shopkeeper's knowledge of malaria treatment. Simultaneously, the shop's locations are recorded with hand-held GPS devices. This approach allows monitoring the shopkeepers' knowledge of malaria treatment, the services and drugs offered, as well as the coverage of shops stocking drugs as a proxy for availability and accessibility.

In a second approach, "mystery shoppers" (simulated clients) buy drugs in local commercial outlets. For this purpose, local villagers are hired and instructed to go to a nearby shop and ask for treatment for fever/malaria on the basis of standard case scenarios.

#### Costing of implementation activities

A financial analysis of the intervention costs will be performed after the interventions have been running for at least two years. A cost-effectiveness analysis will combine measures of effectiveness (see under health impact assessment) and financial costs. For this purpose, a clear distinction is maintained at the level of IHRDC administration between the cost related to interventions and the cost related to research, monitoring and evaluation.

#### Assessing the impact of the introduction of Artemether-Lumefantrine

Tools developed by the ACCESS Programme will be used to monitor prospectively the health impact of the switch in first-line treatment for malaria from SP to Artemether-Lumefantrine. This assessment will be done in the frame of a related but separate project, called "Artemether-Lumefantrine in vulnerable patients – exploring health impact" (ALIVE). It will include monitoring changes in child mortality trends as well as annual community-based cross-sectional studies and an in-depth compliance study in 500 children.

## Progress and results to date

### Community leaders' sensitization and social marketing

**Figure 6:**
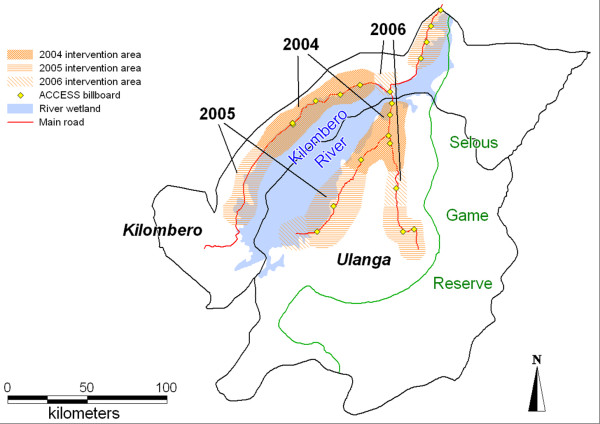
Map of the study area showing intervention areas 2004 – 2006.

Community activities started with the sensitization of community leaders followed by road shows in the 25 villages of the DSS area and in Ifakara town in 2004. The 2005 round covered an additional 56 non-DSS villages in both districts (59%) and by the end of 2006, a total of 114 (79%) villages were reached with both activities (Figure 6). On average 40 community leaders per village (90% of the invited) attended the sensitization meetings (total of over 5,000 in three years) and shared their views and concerns, such as:

"We have seen different health care providers prescribing malaria treatment to patients differently. Some prescribe quinine alone, or SP alone and sometimes quinine and later on SP. We get confused! Which is the appropriate treatment for malaria?" (Participant, Idete village)

"Children, wives and sometimes relatives of the drug shop owners sell in some of the drug shops. We know these people; they have no formal training, only instructions. It is dangerous." (Participant, Igima village).

**Figure 7:**
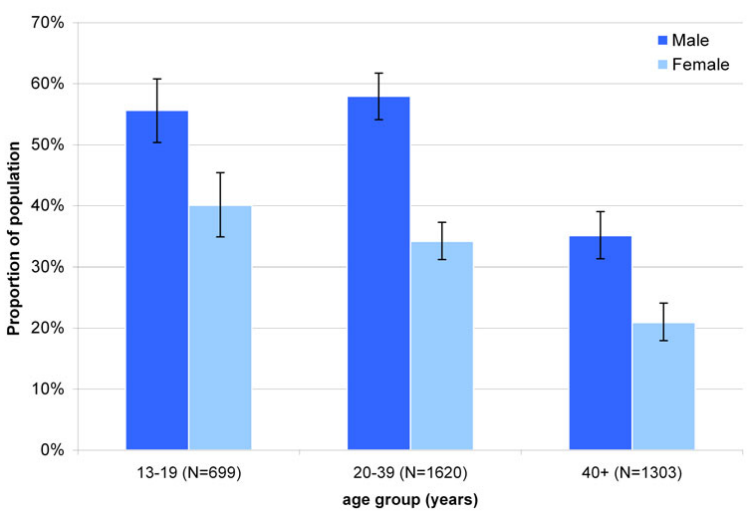
Coverage of social marketing campaign in 25 DSS villages: proportion of the population that has attended an ACCESS road show by age group.

Road shows were generally very well attended. Turn-up varied considerably depending on the size of the village, ranging from few hundred to a few thousand people during big shows such as in Ifakara. In a cross-sectional survey done in 2006 in the DSS area, 39% (95% CI 37.2 to 40.4) of the people mentioned that they had attended an ACCESS road show. Men were 2.2 (95% CI 1.9 to 2.5) times more likely to have attended such a show than women (P < 0.001) and younger people were more often exposed than older (Figure 7). Further, many people had been in contact with or seen promotion materials such as t-shirts and caps (48%), a vehicle displaying ACCESS slogans (46%), or billboards (35%). Community leaders' sensitization meetings reached 16% of the interviewed.

### MCH campaigns

So far, 18 special sessions for pregnant women have been carried out in MCH clinics in the DSS area, one in Ifakara and 28 in non-DSS villages of both districts. In the DSS alone, about 4,700 mothers attended the sessions, representing approximately 28% of all women in reproductive age.

### Health facility intervention

Between November 2004 and April 2005, several refresher training sessions were organised in collaboration with the CHMTs of Kilombero and Ulanga. In Ulanga, 100 (89% of total) clinicians, nurses, medical aids and technicians from rural dispensaries and health centres attended the trainings. In Kilombero, 39 (93% of total) clinicians were trained. The tools for supportive supervision and quality management are currently being developed.

### Accredited Drug Dispensing Outlets

The ADDO programme was targeted at the 32 existing drug stores in Ulanga and 93 in Kilombero District [59,60]. After a preparatory phase of training shop owners and dispensers, setting up or renovating shop infrastructure and a licensing procedure, ADDOs were launched in Ulanga in May 2006 and in Kilombero in July 2006. By end 2006, there were 114 ADDOs operational in Kilombero district and 44 in Ulanga (R. Mbwasi, personal communication).

### Monitoring & evaluation

2004 baseline population of the DSS area was 74,200 people, with a crude death rate of 11.6/1,000 people-years observed (PYO). The probability of dying before reaching the age of one year is 63.9/1,000 PYO and before the age of five 109.5/1,000 PYO. The risk of a fever episode (*"homa kali"*) in the two weeks preceding the interview was estimated at 144/1,000 people between May and August and 119/1,000 between September and December. The risk of a *degedege *episode in the previous two weeks was 12/1,000 people between September and December. Mosquito net coverage during the main cultivation period (and peak malaria transmission season) was high in the field huts, with 93–100% of households having a net in their huts and an average of over 97% of people in the huts sleeping under a net (treated or not) the night preceding the interview [61].

**Figure 8:**
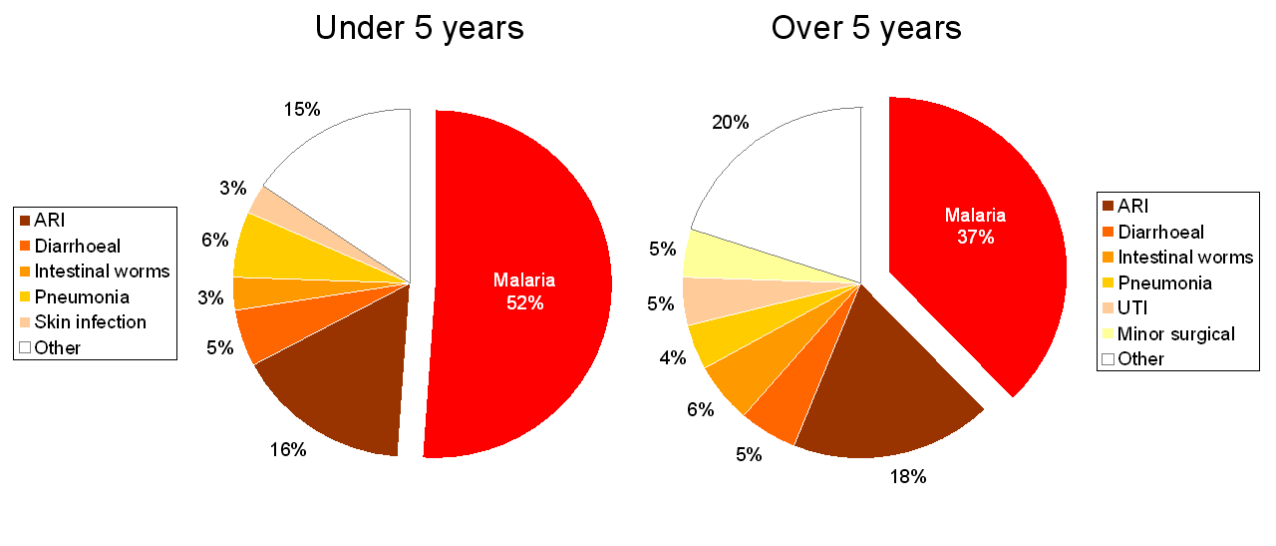
Average monthly out-patients attendance at 16 health facilities in the DSS and Ifakara in 2004 (ARI = Acute Respiratory Infections).

There were seven health facilities in the 13 Kilombero DSS villages and seven in the 12 Ulanga DSS villages in 2004 (one facility per 5,300 people). By 2006, one private dispensary had closed down in Kilombero and a new public dispensary had been opened in Ulanga. The St. Francis Designated District Hospital in Ifakara serves as main referral hospital for all villages in the DSS. On average per month, each of these facilities recorded 240 out-patient visits due to malaria, over 60% of which were children under five. Malaria accounted for 52% of all out-patient visits of children under five and for 37% of all patients over the age of five (Figure 8). In addition to health facilities, there were 30 shops in Kilombero DSS, 13 in Ulanga DSS and 14 in Ifakara offering antimalarial drugs [47].

Focus-group discussions revealed mainly the following malaria-related concerns:

• SP had a bad reputation in Tanzania following media coverage on severe side-effects (Stevens-Johnson syndrome) at the time of its introduction as first-line treatment in 2001[62]. Some people feared SP although they or their children had never experienced severe side-effects, which are known to be rare [63]. People were confused about different SP brand names.

• Modern medical treatment was preferred over traditional medicine and children were treated more quickly than adults. Drug shops were often more conveniently reachable and adults would often buy paracetamol from a shop as first treatment for a fever episode.

• A majority of the people failed to resort to sources of treatment that they otherwise would prefer – such as a hospital. Factors such as cost, absence of trusted medical professionals, unavailability of diagnostic instruments, long waiting time, and distance were mentioned as important obstacles.

These findings, together with national treatment guidelines and information from other projects and surveys were used as basis for developing the behaviour change campaign.

Quality tests of antimalarials (SP, amodiaquine and quinine) purchased from health facilities and shops in 2005 confirmed the existence of sub-standard drugs in the study area. In total 25% of the collected tablet samples did not meet the USP specifications for the amount of active ingredient and were mostly under-dosed. 12% of them contained only minimal amounts of active ingredient. Overall, 24% of the collected SP tablets and 40% of the quinine sulphate tablets were sub-standard. All amodiaquine tablets and quinine injections contained the labelled amount of active ingredient. Sub-standard drugs were found mainly at general and drug shop level and mostly originated from Tanzania and India [57].

## Discussion and conclusion

In order to develop and validate a generic framework on issues related to access to treatment [64], the ACCESS Programme took malaria as an empirical case study. Of course, access issues are also pressing with regard to most other high-burden or neglected diseases in developing countries. By focusing on malaria we chose a poverty-related disease that affects large parts of sub-Saharan Africa in terms of both, disease and economic development, at a time when funding for its control is more readily available than ever before [65].

The Kilombero Valley is an area for which the malaria situation has been particularly well described thanks to numerous research activities [66-69]. The preventive use of insecticide-treated mosquito nets has been advocated through the large social-marketing of the KINET project between 1997 and 1999. It resulted in high levels of ITN ownership and use [29,70]. However, access to prompt and appropriate treatment is still poor. A baseline study in the frame of this programme found that only 14% of young children received an effective antimalarial in the correct dose on the day of illness onset [71]. The aim is, therefore, to expand the successful approach chosen for ITNs to the crucial issue of access to treatment. The main target groups of the interventions are those most at risk in holo-endemic areas such as the Kilombero Valley: young children and pregnant women [21,22,72].

Interventions to improve the complex issue of access to malaria treatment are more likely to be successful if several working approaches are combined. Social marketing applies concepts and techniques used in commercial marketing to prompt behaviour change that benefits the target group [73]. In recent years, it has become increasingly popular in health promotion where it has been proven effective e.g. in promoting the use of ITNs and reducing child mortality [70]. However, care has to be taken that men and women profit equally from the approach – a challenge that has to be tackled by the programme. In the frame of ACCESS, the marketed "product" is the knowledge and awareness of malaria and the concept of treating a malaria episode appropriately. The "price" to be paid by the community is the adoption of the desired care-seeking and preventive behaviour. However, inducement of behaviour change alone is not sufficient; health services which are acceptable and of good quality must be available. Hence, the behaviour change campaign is also a way of empowering the community to demand for good quality health care. Activities to improve quality of health services become central components of the programme.

The major providers of malaria treatment services remain health workers. Their practices are influenced by a variety of factors and environments [20]. The Integrated Management of Childhood Illness (IMCI) strategy adopted by Tanzania is an effective step to improve health worker performance leading to a reduction in child mortality [74] and out-of-pocket expenditures by patients [75]. However, health systems often fail to implement effective guidelines in a sustainable way [76]. The challenge therefore remains to assure adherence to IMCI guidelines and to address factors not directly related to case-management (e.g. motivation or job satisfaction). Multi-faceted approaches including supervision and strengthening of district-level health management are more likely to improve performance [20]. The ACCESS Programme therefore combines training and information with the implementation of a quality-improvement process including strengthening the supportive supervision capacity of the district health management team.

As an alternative to formal health services, antimalarials can be obtained from the commercial sector. Drug shops and general stores are the most important alternative treatment sources for malaria in the study area [58,77]. In an attempt to ensure quality of services, antimalarial drugs sales have recently been banned in general shops. With no alternative sources replacing general shops this policy resulted in a decreased availability of antimalarials in the study area [47]. An alternative approach which has worked well in Kenya would be training of drug vendors [17]. However, current Tanzanian legislation does not allow the selling of antimalarials in general shops. Consequently, any national strategy has to focus on improving the performance of drug stores and their dissemination to underserved areas through the ADDO project.

For the impact evaluation of ACCESS, a plausibility design had to be adopted [51]. Identifying a comparable place as control area would not have been possible and randomization of different areas for intervention would not be feasible within the frame of this programme. Supporting evidence for causally linking an observed impact with the programme's interventions will be obtained through the collection of multiple indicators on intervention delivery, coverage and potential confounders. While the limits of such a design in establishing a causal link are obvious and well known, it needs to be recognized that any large-scale implementation goes through an iterative process of measuring progress and impact while continuously adapting and improving the process. Consequently, the interpretation of results has to take into account contextual changes and external influences. Data from other DSS sites and DHS in Tanzania will be of particular importance in interpreting mortality data and putting them into perspective.

Baseline data demonstrated heterogeneity in the availability of treatment sources, unavailability of medicines and providers and serious quality problems with regard to drugs and services. This supports the basic assumption that there are several inter-linked factors influencing access to effective malaria treatment.

The comparative advantage of the ACCESS Programme is its combination of multiple interventions on different levels of the health system, including a strong evaluation and research component. With this approach, the programme also aims to contribute to the wider debate on access to appropriate health care in developing countries. Based on Penchansky and Thomas' [78] understanding of "access" as the degree of "fit" between the health system and its users, the ACCESS Programme aims at developing a more comprehensive access framework [64]. This can then inform and support public health professionals and policy-makers in the delivery of improved health services, ideally leading to better health and well-being.

## Authors' contributions

MWH was responsible for the baseline surveys of the M&E component and wrote the manuscript in collaboration with the other authors. AS, BO, CL and HM conceived the programme and its components and provided technical support and supervision. AM, CM and NI were responsible for the development and implementation of the interventions. AD, SA and IM were responsible for data collection and analysis for M&E. RN is in charge of the DSS and NI of the overall project management. JDN and RAK were responsible for the IMPACT-Tz household-survey which provided social marketing coverage data. All authors read and approved the final manuscript.
